# Social inequality, scientific inequality, and the future of mental illness

**DOI:** 10.1186/s13010-017-0052-x

**Published:** 2017-12-19

**Authors:** Charles E. Dean

**Affiliations:** Minneapolis Veterans Administration Medical Center, Mental Health Service Line, One Veterans Drive, Minneapolis, MN 55417 USA

**Keywords:** Social inequality, Scientific inequality, Psychiatric diagnoses, The medical model, Biology of mental illness

## Abstract

**Background:**

Despite five decades of increasingly elegant studies aimed at advancing the pathophysiology and treatment of mental illness, the results have not met expectations. Diagnoses are still based on observation, the clinical history, and an outmoded diagnostic system that stresses the historic goal of disease specificity. Psychotropic drugs are still based on molecular targets developed decades ago, with no increase in efficacy. Numerous biomarkers have been proposed, but none have the requisite degree of sensitivity and specificity, and therefore have no usefulness in the clinic. The obvious lack of progress in psychiatry needs exploration.

**Methods:**

The historical goals of psychiatry are reviewed, including parity with medicine, a focus on diagnostic reliability rather than validity, and an emphasis on reductionism at the expense of socioeconomic issues. Data are used from Thomas Picketty and others to argue that our failure to advance clinical care may rest in part on the rise in social and economic inequality that began in the 1970s, and in part on our inability to move beyond the medical model of specificity of disease and treatment.

**Results:**

It is demonstrated herein that the historical goal of specificity of disease and treatment has not only impeded the advance of diagnosis and treatment of mental illness, but, in combination with a rapid increase in socioeconomic inequality, has led to poorer outcomes and rising mortality rates in a number of disorders, including schizophrenia, anxiety, and depression.

**Conclusions:**

It is proposed that Psychiatry should recognize the fact of socioeconomic inequality and its effects on mental disorders. The medical model, with its emphasis on diagnostic and treatment specificity, may not be appropriate for investigation of the brain, given its complexity. The rise of scientific inequality, with billions allocated to connectomics and genetics, may shift attention away from the need for improvements in clinical care. Unfortunately, the future prospects of those suffering from mental illness appear dim.

## The medical model: Has it failed psychiatry?

### Background

In the late 1950s, psychiatry experienced a series of transformations that seemed to hold great promise for the future. As we shall see, these rested primarily on the medical model, a model that emphasized pathological changes in neurochemistry, brain structure, and brain function as the primary causes of illnesses in general―including mental disorders. Implicit in this model is the assumption that each illness has a specific biological cause and treatment, in contrast to the approaches espoused in the psychodynamic and behavioral models of mental disorders. [1, 2].

These transformations included the discovery and use in the 1950s of the first antipsychotic, chlorpromazine, and the first antidepressant, imipramine, each of which spawned a rapidly increasing number of similar drugs, albeit with differing potencies and side-effects. In short order, psychiatrists also had access to a variety of antianxiety and antimanic drugs, including lithium and divalproex [3, 4]. For patients suffering from severe mental illnesses, these agents appeared to provide clear advantages over other biologically-based approaches developed earlier in the twentieth century, including insulin coma, electroconvulsive therapy, and prefrontal lobotomy [5, 6].

Psychopharmacology soon became the dominant force in the treatment of mental disorders, backed by the growing influence of the pharmaceutical industry in the 1970s and 1980s. Academia and industry collaborated in the discovery of biochemically-based mechanisms thought to lie behind the clinical effects of antidepressants, including the catecholamine hypothesis [7], the cholinergic hypothesis [8], and the permissive amine hypothesis [9, 10]. For antipsychotics, the blockade of dopamine receptors seemed critical [11, 12], while gabanergic enhancement seemed important for the effects of antianxiety agents [13]. Lithium was − and is − more complex, but was thought to diminish the activity of catecholamines [14]. Each of these concepts proved to be far too simplistic, but served to spawn hundreds of research projects and the growth of psychopharmacology [3, 4].

At the same time, investigators began to reason backward from the biochemical theories of drug mechanisms to the etiology of the target disorder. For example, the etiology of schizophrenia was based on an excess of dopamine, a marked shift from the dominant psychoanalytic concepts involving the schizophrenogenic mother [15], the struggle of an individual against an abnormal society [16], and the double-bind theory [17], where the individual’s responses to parental expectations were never correct.

It appeared that psychiatry in the 1950s and 1960 was in the early stages of a paradigm shift, marked by a transition from a psychoanalytic/psychodynamic model to a medical, or biological, model. However, we should note that this transition had its roots in psychiatry’s early goal of parity with medicine and surgery [18, 19]. Indeed, the “alienists” of the nineteenth century were often seen as not only isolated from general medicine and surgery [18], but prone to the indiscriminate use of primitive “therapies,” including strait-jackets, blood-letting, hydrotherapy, confinement in rapidly-spinning chairs, or, at the other end of the spectrum, “tranquilizer” chairs. Forced injections of mercury, testicular fluids, or even brain extracts were common [5, 20].

A few of these therapeutic approaches involved a primitive medical model, including Henry Cotton’s theory that infections of the teeth or other internal organs led to psychotic disorders via the transport of toxins to the brain. Cotton and others then proceeded to remove sections of the intestines, the ovaries and uterus, the thyroid, and teeth [5]. Other theories of brain dysfunction soon came into play, with an emphasis on metabolic changes and inflammation, which in turn led to a variety of treatments such as electrostimulation, carbon dioxide therapy, trepanization, and sleep therapy. Nevertheless, evidence of a direct link between the proposed biology of mental disorders and their treatments were ill-defined, and led to considerable criticism by others in medicine, and especially by those in neurology [5, 6].

For the alienists, however, the discovery of the bacterial cause of tuberculosis and other infectious diseases was encouraging, and all the more so in 1913 when treponema pallidum was discovered in the brains of patients with tertiary syphilis (general paralysis of the insane, or GPI), a condition that often resulted in psychosis [6]. Interestingly, there was an ongoing debate among psychiatrists regarding the etiology of GPI, with some claiming that sexual excess and temperamental factors had fostered the development of GPI, and it was therefore a “functional” psychosis. However, treponema pallidum was also present in the brains of syphilitic patients who had not developed GPI [21].

Here at last was substantial evidence of a direct link between a specific causal agent and a specific disease. If this was the case with syphilis, why not mental disorders?

### Genetics and specificity in psychiatry

Another boost to the medical model occurred with the publication of Franz Kallmann’s multiple studies on the genetics of schizophrenia in the 1930s and 1940s. He found an 86% concordance rate for schizophrenia among monozygotic twins [22], while the risk of schizophrenia was 68% in the children of two parents with the illness, suggesting a recessive gene. However, Kallmann’s work suffered from severe methodological problems, which, when corrected in subsequent investigations, resulted in monozygotic concordance rates of 38–48% [23, 24]. With concordance rates of less than 50% in identical twins, questions about non-genetic soon came into play.

Nevertheless, with the discovery of the genetic code, psychiatry in the 1980s embraced the new methodologies, especially after linkage analysis established the genetic basis of Huntington’s disease and cystic fibrosis. Unfortunately, linkage analysis was not successful when applied to psychiatric disorders, nor was candidate gene analysis [25]. By 2006, no genetic variants had been clearly associated with a mental illness, a disappointing result, especially in view of the goals [26] of the Human Genome Project (HGP). These included not only disease prevention [27], but enabling clinicians to “subclassify diseases and adapt therapies to the individual patient,” consistent with the long-sought goals of disease specificity and treatment.

The advent in 2005 of genome-wide association studies (GWAS), capable of genotyping at least 500,000 single-nucleotide polymorphisms (SNPs), appeared to offer even more hope in clarifying the roots of complex psychiatric disorders, and providing a path toward risk prediction, diagnostic precision, and specificity of treatment. By 2009, psychiatry had at least 20 GWAS of schizophrenia, 18 of bipolar disorder, and some 1400 studies of candidate genes. Three years later, 12,000 studies had been published on the genetics of schizophrenia [28], at a cost of some $1.4 billion [29].

While the goals of the HGP have been relatively successful in oncology and other areas, where progress has been made in the prediction and treatment of breast cancer [30], metastatic melanoma [31], the diagnosis and treatment of hepatitis C [32], and others too numerous to mention here, has there been any comparable progress in psychiatry?

The answer appears to be no. Instead, GWAS have undercut the concept of disease specificity by demonstrating significant genetic overlap across multiple mental disorders, including schizophrenia, bipolar disorder, major depressive disorder, autism, and attention deficit hyperactivity disorder [33]. A major review of the genetic architecture of psychiatric disorders by Patrick Sullivan and colleagues [34] noted that the rare but potent variants found in schizophrenia and bipolar disorder also increased the risk of developmental delay, epilepsy, head size, somatic dysmorphism, and extremes of body mass. While the odds ratios (ORs) for these structural variations ranged from 2.1 to 20.3, the frequency in cases ranged from 0.0006% to 0.0124%, making clinical usefulness highly questionable. Similarly, the ORs in schizophrenia averaged 1.14, very similar to the average OR of 1.10 in another GWAS involving over 16,000 patients and 14,000 controls [35]. Put another way, these studies resulted in a 10–14% increase in the risk for schizophrenia.

In a widely-cited GWAS [36] of almost 37,000 cases and 113,000 controls, the authors found 108 schizophrenia-associated independent genetic loci, but these loci accounted for only 3.4% of the variance. However, if one combines the genetic loci, one can generate a polygenic risk score (PRS). In this study, the PRS increased the liability score to 7%. Nevertheless, the authors specifically stated that the sensitivity and specificity of the PRS did not permit its use as a predictive test―although others claimed that combining genetic variants resulted in good case-control discrimination [37]. Indeed, the current trend is to further expand the possibilities by combing gene clusters with symptom profiles [38] or brain circuits [39].

While we have focused on schizophrenia, the results have been similar in other conditions. A mega-analysis of GWAS in major depression [40] failed to identify any “robust and replicable findings,” while in autism spectrum disorder [41], small effect sizes were common, despite the fact that common variants represented the greatest proportion of the risk. Even in the case of rare de novo variants, their contribution to the total variance was low.

There is growing agreement that even when we aggregate data from GWAS in schizophrenia, the results explain only a “tiny proportion” of disease familiality [42]. In addition, the list of potentially important risk loci has increased dramatically, rising from 3 in 2009 to 17 in 2012, and to 108 in 2014 [43]. In an interview [44], Patrick Sullivan noted in 2014 that as many as 6000 to 10,000 independent SNPs and 1000 genes may contribute up to one-third of the total variance in schizophrenia. However, as Kendler and O’Donovan [43] have observed, “Long lists of weak risk variants on their own are of limited scientific importance.”

With regard to specificity and clinical applicability, we should note the following:

- Schizophrenia can occur in the absence of the most robust genetic findings, and common variants are associated with a range of disorders [45, 46].
- There is a full spectrum of mental disorder among children of parents with mental disorders [47, 48].
- The same mutation may lead to different phenotypes, while mutations in different genes in the same or related pathways may lead to the same disorder; thus, there is “vast” genetic heterogeneity in human disease [49].

### Have imaging studies supported the specificity of mental disorders?

Once again, the answer appears to be no, despite the advent of multiple imaging modalities ranging from CT scans to fMRI and diffuse tensor imaging. As of 2008, more than 7000 MRI brain scans of patients with bipolar disorder had been performed but with widely varying results. That being the case, a meta-and mega-analysis of 98 studies found only non-specific changes [50]. While deficits in working memory have long been proposed as a marker of frontal lobe dysfunction is schizophrenia, an analysis of 30 relevant imaging studies found that half have shown hypofrontality, a few have found no differences, while the remainder noted hyperfrontality [28, 51]. A recent meta-analysis of 80 MRI studies of psychoses published between 1976 and 2015 found no diagnostic or prognostic biomarkers [52]. With regard to depression, a meta-analysis of studies of aberrant brain function during cognitive and emotional processing failed to find any statistically significant evidence of convergence across 59 studies [53].

In a 2015 editorial [54], Sommer and Kahn observed that neuroimaging had been extremely productive in schizophrenia research, yielding “a wealth of data on brain abnormalities,” but what of their clinical relevance? Can imaging be used to assist in diagnosis, risk prediction, and outcome? While the authors were cautiously optimistic about the use of PET scanning and magnetic resonance spectography in assessing treatment outcome, they also noted that most neuroimaging studies are characterized by extensive overlap between cases and control groups.

In summary, genetic and imaging studies have no doubt added to our understanding of brain structure, function, the delineation of brain circuits, and the biology of psychotropic drugs, but they have provided little evidence of support for the specificity of disease and treatment, or for clinically useful biomarkers [28]. The lack of clinically useful results [55] may rest, at least in part, on their “lack of biochemical precision and neuropathological validation,” which has resulted in a significant gap between research results and their translation into clinical usefulness [56]. Nevertheless, these modalities remain the principal means by which the National Institutes of Mental Health will advance the goals of precision diagnosis and precision medicine [57, 58].

## The search for diagnostic specificity and reliability: A contradiction?

We noted earlier the developments in infectious diseases that helped boost the quest for specificity of diagnosis in psychiatry, a quest that gained additional emphasis with the work of Emil Kraepelin, who in 1899 postulated a boundary between dementia praecox (schizophrenia) and mania [59, 60]. The rise of psychopharmacology in the 1950s lent additional urgency to the need for a better diagnostic system, particularly given the need for better research methodology [3, 6].

At the same time, the rise of psychoanalysis and the psychodynamic model undermined the concept of disease and treatment specificity, a clash noted by Roy Grinker, who wrote in 1964 that psychiatry was riding madly in all directions [61]. The basic science of psychiatry was held to be a comprehensive understanding of the unconscious [62], while the fundamental tools of the psychiatrist were held to be psychotherapy and the dyadic therapeutic relationship [63], all of this in contrast to the rapid rise of psychopharmacology and neurochemistry [3, 4, 7, 11].

By the 1960s, few people attended the American Psychiatric Association’s sessions on diagnoses [64], despite a 1962 amendment to the Food, Drugs, and Cosmetics Act requiring that prescription drugs were to be used for the treatment of categorical (specific) diseases [3]. Yet psychiatric diagnoses were not only ill-defined but based almost entirely on clinical consensus―a problem noted by the neo-Kraepelinians at the Washington University in St. Louis, who had begun to question the role of psychoanalysis in psychiatry.

In 1970, Eli Robins and Samuel Guze insisted that the validity of any psychiatric diagnosis rested on its usefulness in prediction, pathogenesis, outcomes, and response to treatment [65]. In general, the concept of validity refers to the degree to which a mental disorder correlates with external validators, including outcomes, prediction of treatment response, family history and neurobiological markers. The emphasis on validity led to the publication of the Feighner diagnostic criteria in 1972 [66], a system that used family and outcome studies as the basis for inclusion, rather than clinical consensus. The authors found only 14 diagnostic categories. The system also required the use of a diagnostic checklist of specific symptoms and exclusion criteria for each disorder (“operationalized criteria”), rather than relying on a clinical narrative.

The Feighner criteria were followed the Research Diagnostic Criteria (RDC) in 1978 [67], a system that also used operationalized criteria, but which expanded the list of categories to 28. In contrast to the Feighner system, the RDC included studies on diagnostic reliability, i.e., the degree to which clinicians agree on a diagnosis. Although reliability seems a worthy objective, the problem is that there is “no empirical basis from which to argue that a system on which people agree has greater scientific validity…” [68]. In fact, increases in diagnostic reliability have not been accompanied by increases in validity [69].

With the publication in 1980 of the DSM-III [70], the neo-Kraepelinian focus on validity largely disappeared, and was replaced by clinical usefulness, reliability, and inclusiveness. Disease specificity took a back seat, with the foreword to the DSM-III noting that “there is no assumption that each mental disorder is a discrete entity with sharp boundaries (discontinuity) between it and other mental disorders, as well as between it and No Mental Disorder.” This led to an expansion of diagnosable disorders to 256, a veritable epidemic. In addition, there was no coherent rule for including or excluding categories, a process I characterized as “diagnostic democracy,” not science [71, 72]. However, DSM-III also noted that some categories were excluded due to lack of supporting evidence, but “most” categories lacked such evidence, leaving the rationale for inclusion murky at best.

### Clinicians and a dimensional approach to treatment

Despite the shift to an all-inclusive poorly-validated diagnostic system in DSM-III and subsequent editions, experts such as Nancy Andreasen, in her book, The Broken Brain [1], continued to insist in 1984 that “each different type of illness has a different specific cause.” Psychiatrists should therefore treat the specific disease with disease-specific medications, an approach typical of a categorical diagnostic system that implies the existence of discrete disease entities. As we have seen, the DSM-III appeared to disavow this approach, although investigators were usually required to use DSM-III criteria in their research, although they were most often engaged in attempts at separating one illness from another. Yet DSM-III seemed to embrace a dimensional approach that did not draw boundaries between and among disorders.

The current goals of the NIMH appear to encompass an admixture, using a symptom-based dimensional approach, but with the goal of defining specific disorders in terms of neural circuits that will eventually allow the delineation of specific patient subtypes treated with precision medications [39, 57, 58].

Have how clinicians responded to these shifts in goals? The data indicates that clinicians, and to some extent, the FDA, have already moved to a dimensional, symptom-based approach [72–74]. For example, the FDA has been busy approving individual drugs for a host of disorders, with sertraline approved for at least six individual disorders, while atypical antipsychotics have been approved for schizophrenia, and in some instances, for bipolar mania, bipolar depression, major depression, and autism. Allegedly specific disorders such as major depression can be treated with multiple classes of antidepressants, antipsychotics, vagus nerve stimulation, transcranial magnetic stimulation, and ketamine, not to speak of cognitive behavioral therapy. Interest is growing in the use of hallucinogens [75], whole body hyperthermia [76], and even opiates [77] for treatment-resistant major depression.

The move to a symptom-based approach is reflected as well in the growing use of drugs used off-label, with the off-label use of antipsychotics rising from 6 million treatment visits in 1995 to almost 17 million in 2006, at a cost of some $6 billion [78]. In a study of 280,000 veterans, 60% had no record of an FDA-approved indication [79]. In Germany, 63% of antipsychotic use was aimed at sleep disorders, neuroses, and dementia [80], prompting the authors to recommend a return to the use of “tranquilizer” as the more appropriate label. In a study of antidepressants, about 50% of prescriptions were written for off-label use [81, 82].

### The rise and fall of the neo-Kraepelinian school of psychiatry

While the Washington University school of neo-Kraepelinians was instrumental in reorienting psychiatry from a psychodynamic/psychoanalytic model to a medical model, there was a paradox, in that Kraepelin himself began to reverse course from his earlier distinction between manic-depressive insanity and dementia praecox.

That distinction had been the basis for the “Kraepelinian dichotomy” that was so influential in Western psychiatry, as noted by Craddock and Owen in 2005 [83]. However, the authors wentt on to note that genetic and family studies were undermining the dichotomy, consistent with the results just reviewed. Yet Kraeplin himself [84] had begun shifting his original position as early as 1899, when he observed that there are (a), no pathognomic symptoms in insanity, (b), the same clinical presentation can be found in otherwise divergent disorders, and (c), that we cannot distinguish manic-depressive illness from schizophrenia, and (d), that there are no infallible criteria or sharp boundaries between health and disease.

The reader will no doubt notice that Kraepelin’s observations are not only similar to those found in the DSM, but are remarkably similar to the results of the contemporary genetic and family studies described by Craddock and Owen [83], and updated in this paper. Nevertheless, psychiatry persists its search for specificity, but in a modified form, such that classic DSM disorders are to be replaced by dysfunctional neural circuits [57].

### Clinical care in psychiatry: Progress, stalemate, or worsening outcomes?

In 2009, Thomas Insel, then director of the NIMH, wrote [85] that genomics and imaging studies have “…not yet impacted the diagnosis and treatment of the 45 million Americans with serious or moderate mental illness each year.” In the case of schizophrenia and bipolar disorder, Insel also noted that there is little evidence indicating that the prospects for recovery have changed during the past century, nor is there evidence for a reduction in morbidity, mortality, or disability.

Indeed, there is considerable evidence [86] that matters have worsened since the 1970s, with the median standardized mortality ratios (SMRs) for schizophrenia rising from 1.84 in the 1970s to 2.98 in the 1980s, and to 3.2 in the 1990s, despite the advent of atypical antipsychotics and other interventions. The rise in SMRs clearly contrasts with the 43% decrease in the SMR found in the general population during the years 1969–2018 [87], with the exception of the non-Hispanic white population [88]. In a study covering the years 2001–2007, Olfson, et al. [89] found an SMR of 3.7 in adults with schizophrenia. The mortality gap in first-contact psychotic patients also widened during the years 1965–2007 [90], and now includes those with anxiety disorders [91].

In a 2017 study [92] of schizophrenia and bipolar disorder covering the years 2000–2014, the authors found falling rates of all-cause mortality (cardiovascular events, suicide), but the hazard ratios (HRs) increased when compared with the matched general population. Indeed, the HR for schizophrenia was 2.08 higher than the general population, while in bipolar disorder, the HR was 1.77.

The HRs were somewhat lower than in previous studies, perhaps reflecting the multiple campaigns aimed at reducing mortality rates [92]. However, the authors noted that the results indicated increasing inequalities in health care, although a study [93] in Denmark―noted for its free health care system―found that the average age of death in patients with schizophrenia was 62.2 years, compared with 73.4 years in the general population. When examined over time, there was a steady fall in the age of death in schizophrenia, in contrast to a steady rise in the age of death in the general population.

In contrast, several studies in Finland failed to find an increase in mortality over time [94, 95], but in a meta-analysis of studies carried out in 29 countries in six continents [96], the pooled relative risk for mortality in patients suffering from psychosis, mood disorders, and anxiety disorders was twice that of the general population. The authors noted that studies with a baseline starting in the 1990s demonstrated a stronger effect than did those begun prior to 1970, again indicating that matters had worsened over the decades.

In addition to rising mortality rates, improvement rates in schizophrenia fell to a mean of 36.4% in the years 1986–1992 [97], a considerable drop from a peak of 48.5% in 1956–1985, and comparable to the mean of 27.6% in 1895–1925, decades before chlorpromazine was introduced in the 1950s. However, the authors emphasized that the fall-off from 1986 to 1992 may have been, at least in part, secondary to the use more restrictive diagnostic criteria, but they neglected to note the historic rise in income inequality that began in the 1970s―a topic we shall discuss in the next section. Consistent with this, we should note that the odds of any adverse outcome in schizophrenia―including self-harm, illicit drug use, and criminality―steadily increased during the years 1972–2009 [98].

Similar problems have been documented in depression, which in 1964 was seen by Jonathan Cole at the NIMH as having the best possibility for recovery, with or without treatment [99]. Ten years later, he noted that a return to the premorbid state was common, and that depression usually occurred in the form of an acute, single episode [100], although melancholia was an exception.

This optimistic view of depression was undercut by several long-term follow-up studies that found increasing rates of depression in successively younger birth cohorts across some countries [101–103], but not others [104, 105]. In the United States, however, the prevalence of major depression doubled from 1991 to 1992 to 2001–2002, and did so among all age groups, whites, African-Americans, and Latinos [106].

In addition, the optimistic view of treatment was overshadowed by the results of a thirty-year follow-up of patients enrolled in the Collaborative Depression Study (CDS) that enrolled patients from 1978 to 1981 [107]. In marked contrast to Cole, the CDS found that depression is a recurrent illness, with a relapse rate of 40% at 2 years, 60% at 5 years, and 85–91% at 30 years. The median time to recovery was 30 weeks. Twenty percent failed to recover at two years, no matter the treatment. Indeed, the number of treatments aimed at depression had increased dramatically since the advent of imipramine in the late 1950s [4].

Given the increase in the prevalence and chronicity of depression, it isn’t surprising to find that during the years 1992–2002, the suicide rate rose by 35% in adults ages 35–64, and by 60% among women ages 60–64 [108]. A significant increase also occurred among non-Hispanic white males ages 45–64, accompanied by a loss of jobs and high rates of alcoholism [88].

Unfortunately, it appears that people with mental disorders have not been able to take advantage of the improvements in health care provided to the general population. For example, in a 24-year national register study covering the years 1987–2010, Westman, et al. [109] found that patients with schizophrenia ages 15–59 not only had a a 5-fold increase in acute myocardial infarction, but were hospitalized less frequently and died more often than those in the general population. Remarkably, the number of excess deaths from cardiovascular disease exceeded those from suicide.

The risk from cardiovascular disease is not confined to schizophrenia, but extends to those with major depression and bipolar disorder [110]. Even after adjusting for confounders, this group of patients have a 53% increase in risk for cardiovascular disease, with about 10% having at least one cardiovascular disease at age 50. The authors also called attention to the increased pool of people at risk, given the wide-spread use of atypical antipsychotics with their metabolic side-effects.

If we expand our discussion to changes that occurred in the United States on the county-wide level during the years 1980–2014, we find that the over-all mortality rates associated with mental health and substance abuse disorders rose by 188%, and by as much as 1000% in clusters of counties in the southeast and midwest [111].

## Psychiatry’s blind eye to the role of socioeconomic inequality in the etiology and course of mental illness

### Income inequality, social deprivation, and mental illness

Clearly, the increasingly dismal outcomes found in many psychiatric disorders cannot be attributed to a lack of funding for genetics and imaging studies. Nor can a lack of therapists, the number of which rose by 80-fold in the U.S from 1940 to 2010, while the population only doubled [112]. We should also note that spending on personal health care 1996–2013 increased substantially in the United States, reaching $1.2 trillion by 2013, with an estimated annual increase of 3%–4% [113]. Indeed, spending on depressive disorders reached $71 billion in 2013, ranking 6th among 155 medical conditions, and surpassing funding aimed at anxiety disorders ($29 billion), ADHD ($23 billion), and schizophrenia ($17.6 billion).

That being the case, we should look elsewhere for answers to the downward shift in outcomes for those with mental disorders―a surprising development given the intense focus on the biology of mental disorders and the availability of multiple classes of psychotropic agents. In addition, psychiatry had placed more emphasis on psychotherapeutic approaches, including cognitive behavioral therapy, social skills training, supportive work therapy and the like [114].

#### What happened?

First, we should observe that in 1993, states began to cut funding for clinical care, despite a higher total outlay for mental health [115], but the increase was largely directed toward the criminal justice system and disability payments. By 1993, the number of psychiatric beds had fallen from 34/100,000 population to 22/100,000 population, a 34% reduction. This gave the United States the dubious distinction of having fewer psychiatric beds than found in all but 4 countries in the Organisation for Economic Cooperation and Development [116]. In some states, the numbers were even worse. In North Carolina, for example, the number of beds fell to 8/100,000 [117], while funding for community care fell by 20%. The same pattern was found in the state of Washington, where beds fell by 36% while funding was dropped by $90 million, undercutting the notion that patients would be cared for in the community. Making matters even worse, the number of beds continued to fall during and after the 2008 financial crisis [117].

Second, and perhaps more importantly, psychiatry appears to have neglected the impact of the dramatic change in the economic status of the middle and lower income classes in the United States, a change that began in the 1970s [118, 119]. Indeed, during the decades spanning 1973–1993, some $255 billion in wealth shifted from the middle quintile to the top [119]. This shift continued in the years 2007–2010 where the median household wealth fell by 40% [120]. Since 2008, the ranks of the middle class have fallen by 20%. Most workers in the United States have seen little, if any, increases in real wages since the 1970s [120].

The consequences have been severe, with almost 46 million people living in poverty, compared with 37 million in 2007 [121]. Since 2008, the number of homeless children attending public schools rose by 72% [122]. Those qualifying for disability payments due to mental illness increased from 1 in 184 to one in 75 by 2007 [123]. During the past 30 years, the prison population in the U.S. increased by 400% [124], with the rate of mental illness among prisoners rising from 5% in the 1970s to 20–40% currently [125], making the prison system the largest caretaker of the mentally ill in the U.S. The Cook County sheriff, Thomas Dart, observed that the mentally ill have nowhere else to go, due to the closure of mental health facilities [126].

### Inequality and mental illness

Wilkinson and Pickett [127] have shown a close correlation between levels of inequality and rates of mental illness, substance abuse, infant mortality, child well-being, teen-age pregnancies, and shorter life-spans, whether in countries or across states in the United States. Societies with the highest rates of inequality have five times the rates of imprisonment, and six times the rates of obesity. In the 1980s, the rate of social mobility began to decline, trapping people in the lower income groups.

Even subjective social status has been inversely related to mental disorders in 14 of 18 countries; this persisted after adjustment for objective indicators of social status [128]. Poverty itself ―aside from stress― can impair cognitive functioning [129]. This in turn impairs one’s ability to focus on longer-term goals such as obtaining a good education and adopting healthy behaviors [130].

The importance of unhealthy behaviors (smoking, obesity, inactivity) behaviors has been demonstrated by their moderating effect on the association between all-cause mortality and lower social status [131], an issue clearly relevant for people with major mental disorders. Indeed, a recent study demonstrated that differences in life expectancy for those in the lowest income quartile were significantly correlated with health behaviors, but not access to health care, income inequality, or conditions in the labor market [132]. However, inequality in health care increased with time, and higher incomes were correlated with increased longevity across the income distribution. The importance of education on life expectancy was noted by Meara, et al. [133], who found that changes in mortality and life expectancy during the years 1981–2000, were correlated with levels of education.

Levels of inequality among mothers have led to serious consequences, including higher rates of smoking, poorer pre-natal care, poorer nutrition, and higher rates of stress and violence [134], all of which may have long-term consequences for the offspring.

It seems clear that socioeconomic adversity significantly increases the risk of mental illness. This is not a new development. The National Comorbidity Survey [135] in 1994 found that the risk of 3 or more mental disorders in the same person was doubled by low levels of income and education, while urbancity increased the risk by 20%. Similarly, Peen, et al. [136] found in 2010 that current city residents had a 20% increase in the risk of mood disorders, and a 21% increase in the risk for anxiety disorders. The risk for schizophrenia doubles for those born and raised in cities [137], while a study in the East End of London found that population density, income inequality, and deprivation increased the risk for non-affective psychotic disorders by 18%j, 25%, and 28% respectively [138]. Migration alone carried a 3-fold increase in the risk for schizophrenia, considerably greater than the risk associated with winter birth, obstetric complications, or genetic factors [139]. Note that the risk from genetic factors is, at best, 35% compared with 250% for environmental factors [140].

The authors of a recent study [37] on the prediction of risk derived from the polygenic risk score (PRS) noted that the OR for psychosis was similar to that found from social disadvantage at one year prior to disease onset. The further observed that the PRS may never be powerful enough for risk screening, and recommended that future studies examine the influence of migration, socioeconomic differences, physical health, and quality of life.

Others have recommended that studies on gene expression should focus on changes during periods of vulnerability, since gene expression changes continually over the life span [141]. However, such data is virtually absent from GWAS, which are cross-sectional and almost always ignore socioeconomic factors.

### Psychiatry’s response to failure: Scientific elitism.

Despite the failure of genetics and neuroimaging to improve clinical care, and despite evidence indicating that socioeconomic factors play a significant role in the genesis, persistence, and worsening outcome of mental disorders, the leadership in psychiatry remains focused on the long-standing medical model aimed at development of precise diagnoses, precision medicine, and the elucidation of specific patient subtypes [85, 142–144]. Progress in these areas is proposed to flow from new and data-rich approaches to brain mapping and very large-scale genetic studies. The new head of the NIMH, Josh Gordon, has made this explicit [145], noting that his priorities are neural circuits, computational psychiatry, and, fortunately, suicide prevention. In this schema [146], psychiatrists will be known as “circuit psychiatrists,” a slight change from a 2005 suggestion [143] that psychiatrists will be known as “clinical neuroscientists.”

No one should underestimate the costs of the new brain mapping and genetic studies. The Human Brain Initiative will require $4.5 billion spread over a decade [147], while the European Commission for the Human Brain Project will invest some $1.3 billion on a supercomputer simulation that aims to model everything known about brain structure and function [148]. The Allen Institute for the Brain Sciences [149] will invest $300 million during the first four years on a project aimed at mapping the mouse cerebral cortex. Another $500 million will be devoted to the development of precision medicine [150].

The U.S. Congress recently proposed the twenty-first Century Cures Act [151] that includes $1.4 billion for the Precision Medicine Initiative, $1.6 billion for the Brain Research through Advancing Innovative Technologies, $1.8 billion for the “cancer moonshot,” and $30 million for research on regenerative medicine. Other projects include the 10-year Japan Minds Project at $300 million, and the 10-year Korea Brain Project at $500 million [152–154].

The billions proposed for these projects has raised a number of concerns, in addition to their enormous costs. For example, more than 750 neuroscientists in Europe signed a letter to the European Commission criticizing the move by the project director of the Human Brain Project to decrease the focus on fundamental neuroscience, and instead emphasize human imaging, atlases, and brain simulation [154, 155]. This led to the establishment of an independent commission that not only supported the criticisms, but recommended changes [156].

The proposed International Brain Initiative has also come under scrutiny, given differing research priorities among countries, and the requirement that each country contribute $300 million to the project, thereby excluding poorer nations [157]. The latter has raised concerns over the development of inequality in science [158]. Indeed, Nature [159] devoted a special issue to the subject, asking “Is science only for the rich?” A number of writers then proceeded to outline the difficulties in finding support in poorer countries for those interested in a scientific career.

Not surprisingly, others have questioned the focus on the technology paradigm [160] and the search for ever-larger data bases, including the million-person megastudy aimed at developing precision medicine [161]. In the meantime, funds for research aimed at improving clinical care have languished [162]. Holmes, et al. have noted that funding for research into psychological treatments hovers around 15% of the money devoted to mental health research, despite substantial evidence for efficacy [163].

Regardless of the paradigm and the allocation of funds, we still have the problem of a deeply flawed diagnostic system. Remarkably enough, even Robert Spitzer, the prime mover of the DSM-III, admitted in an interview that the DSM is not scientific [164], while Nancy Andreasen wrote that it is not suitable for research, given its lack of validity [165].

The NIMH has responded to these criticisms by initiating a shift from DSM-defined diagnoses to the Research Domain Criteria (RDoC) [57, 58]. This system incorporates 7 units of analysis ranging from genes to self-reported symptoms and behaviors. The RDoC is aimed at cutting across DSM categories, and thus has the markings of a dimensional diagnostic system. However, it is also aimed at identifying disorders of specific neural circuits and their relationship with clinical phenomena, with the ultimate goal of advancing precision medicine for mental disorders.

There are two problems here, one being the remarkable plasticity and biological heterogeneity of neural circuits [39]. That being the case, how does one develop precision medicines aimed at neural circuits that are subject to constant changes stemming from mutations, stress, and infections [39], not to speak of social status [166]. Nor is it clear how a single drug can therapeutically alter a circuit that no doubt will be comprised of tens of millions of neurons and billions of synapses? After all, Insel, et al. [167] have stated that only one mm3 of brain tissue contains at least 80,000 neurons and 4 million synapses. Nevertheless, the authors clearly noted [57] that the critical test for the RDoC is “…how well the new molecular and neurobiological parameters predict prognosis or treatment response,” which brings us back to the Robins and Guze stance of 1970 [65].

## Conclusion

There is no evidence that the past 50 years of neuroscientific research in psychiatry have led to any advances in treatment comparable to what we have seen in the diagnosis and treatment of breast cancer, hepatitis C, malignant melanoma, and some forms of leukemia, to name only a few. In contrast, not one psychotropic drug has been developed based on genetic studies, and we have no replicable or clinically useful biomarkers. Worse, no advances have been made in extending the life span or quality of life in those with mental disorders. Instead, outcomes have worsened in schizophrenia and other disorders, and rates of suicide have risen.

The collective failure of our efforts appears to rest on a complex and interlocking web involving:

1. The historic goal of parity with medicine and surgery, a goal that led us to emphasize specificity of diagnosis and treatment, despite growing evidence that the complexity of the brain requires a dimensional diagnostic framework. We now have the RDoC, a dimensional system, but one still aimed at the discovery of specific neural circuits and precision medicine, despite the plasticity and complexity of neural circuitry.
2. The search for homogeneity of patient populations in genetic and imaging studies. This led us to minimize or neglect the evidence for pervasive co-morbidity [168]. For example, in a GWAS of over 36,000 cases of schizophrenia [36], there was no discussion of co-morbid diagnoses or whether they were ascertained. This does not reflect reality.
3. It also appears that geneticists like to pretend that the 15,000 subjects in a GWAS have no significant socioeconomic differences, levels of trauma, or quality of life, again a failure to reflect reality.
4. The failure to recognize, until recently, that there are marked levels of heterogeneity within a single DSM diagnosis. For example, a recent study of major depressive disorder found some 1000 unique clinical profiles [169].
5. A concomitant failure to recognize the impact of socioeconomic status on the genesis and maintenance of mental disorders [170]. This became even more important with the marked increase in levels of income inequality in the United States that began circa 1970.
6. The failure to develop strategies aimed at longitudinal tracking of genetic and imaging studies in order to measure the effects of time, epigenetic changes, stress, poverty, chronic illness, immigration, migration, and other environmental changes.

Finally, I suggest that the leadership in psychiatry should consider a more equitable balance in funding for clinical research, as opposed to the extremely expensive projects devoted to brain imaging and ever-larger genetic studies. An example: the Psychiatric Genomic Consortium now aims to enroll 100,000 cases for as many as 11 mental disorders [171].

While I applaud the transition from the DSM diagnostic system to the RDoC, the latter appears to rest on largely unproven assumptions, poorly validated components, and a high level of reductionism [172, 173].

While we gather massive amounts of data on neural circuits, life in the clinic may become more difficult, as patients face the prospects of additional job loss and financial stress secondary to higher levels of income inequality and the loss of jobs stemming from automation and advances in robotics. Unfortunately, the future for those with mental illness appears dim.

## Abbreviations

CDS: Collaborative Depression Study
DSM: American Psychiatric Association Diagnostic and Statistical Manual
FDA: Food and Drug Administration
GWAS: Genome-wide-association studies
HRs: Hazard ratios
NIMH: National Institutes of Mental Health
ORs: Odds ratios
PRS: Polygenic risk score
RDC: Research Diagnostic Criteria
RDoC: Research Domain Criteria
SMRs: Standardized mortality ratios
SNPs: Single nucleotide polymorphisms
